# Ommochrome Wing Pigments in the Monarch Butterfly *Danaus plexippus* (Lepidoptera: Nymphalidae)

**DOI:** 10.1093/jisesa/ieac076

**Published:** 2022-12-23

**Authors:** Kyri A Forman, Craig D Thulin

**Affiliations:** Department of Chemistry, Utah Valley University, Orem, UT 84058, USA; Department of Chemistry, Utah Valley University, Orem, UT 84058, USA

**Keywords:** monarch butterfly, wing, pigment, ommatin, xanthommatin

## Abstract

Monarch butterflies (*Danaus plexippus*) use bright orange coloration to warn off predators as well as for sexual selection. Surprisingly the underlying pigment compounds have not been previously characterized. We used LCMS and fragmentation MS (including MSMS and MS^n^) of extracted pigments from nonmigratory summer-generation female monarch forewings to identify and provide relative quantitation of various orange pigments from *D. plexippus*. We observed seven ommochrome pigments, with xanthommatin and decarboxylated xanthommatin being the most abundant followed by xanthommatin methyl ester. Among the seven pigments, we also observed molecules that correspond to deaminated forms of these three amine-containing pigments. To the best of our knowledge, these deaminated compounds have not been previously discovered. A seventh pigment that we observed was α-hydroxyxanthommatin methyl ester, previously described in other nymphalid butterflies. We also show that chemical reduction of pigment extracts results in a change of their color from yellow to red, concomitant with the appearance of dihydro-xanthommatin and similarly reduced forms of the other pigment compounds. These findings indicate that monarchs may employ differences in the redox states of these pigments in order to achieve different hues of orange.

The monarch butterfly *Danaus plexippus* is widely known for its bright orange color and annual migration. Recently listed as endangered by the International Union for Conservation of Nature (IUCN Red List’ 2022), it is perhaps the most familiar of the North American butterflies; an iconic pollinator (Chan 2017), that has even traveled into space (NASA 2010). Monarchs use their bright coloration both to warn predators of a distasteful poison as well as to attract mates in sexual selection (Davis 2014). It is therefore somewhat surprising that the pigments involved in this orange wing coloration have not been characterized chemically.

Butterflies use a wide variety of pigments for wing colors. Within the Lepidoptera, many different families specialize in particular types of pigments such as papiliochromes in Papilionidae, pterins in Pieridae, and ommochromes in Nymphalidae (Shamim et al. 2014, Futahashi and Osanai-Futahashi 2021). Ommochromes are biosynthesized from tryptophan and are some of the more common pigments found in insects. They can range from brown to yellow, orange, or red. Ommochromes include the higher molecular weight ommins, (see Figon and Casas 2019), the ommidins (Pinamonti et al. 1973), and the ommatins, which include the well-known phenoxazine-pyridine compound xanthommatin, the structure of which was elucidated by Adolf Butenandt in 1954 (Butenandt et al. 1954). In one report, nine different ommatin pigments were found in the orange regions of female *Elymnias hypermnestra* butterfly wings (Panettieri et al. 2018). The pigments were identified as xanthommatin and related compounds including decarboxylated xanthommatin and some novel ommatins. In another genus, the common buckeye *Junonia coenia* has a seasonal polyphenism with two differently pigmented forms. Those reared in long-day conditions exhibit a tan color (*linea*) and contain mainly xanthommatin and dihydro-xanthommatin, while those reared under short-day conditions showed a red coloration (*rosa*) which comes from ommatin-D (Nijhout 1997).

The pigment xanthommatin takes on different redox-dependent color states, as seen in squids, where the reduced form (dihydro-xanthommatin) is seen as red and the oxidized form is yellow (Williams et al. 2016). The use of redox control of xanthommatin to change coloration is also seen in insects, as was reported in various dragonflies (Futahashi et al. 2012). In an example of technology imitating life, Kumar and collaborators have built electrochromic displays based on this redox-dependent difference in the color of xanthommatin (Kumar et al. 2018). Redox dependence of color has not previously been reported in butterflies.

## Materials and Methods

### Insects and Pigment Extraction

Nonmigratory summer-generation specimens of *D. plexippus* were collected from wild local populations, and insects used in the study were female either wild-caught or F1 generation from captive breeding of the wild-caught specimens.

Pigments were extracted from ~2 mg pieces dissected from the orange sections of forewings by vortexing 10 s in 200 µL methanol with 0.5% hydrochloric acid, followed by centrifugation at 14,000 × *g* for 5 min with the supernatant being carefully removed for study (undissolved material being discarded). Extracts were then concentrated to ~20 µL in a centrifugal vacuum concentrator and 100 µL of 1% formic acid was added. Pigment extractions were done in blue/green colored tubes wrapped in foil to prevent light exposition. Ambient room lights were turned off during the extraction process.

### Mass Spectrometry

Mass spectrometry was done using an LCQ Fleet (Thermo Fisher) quadrupole ion trap instrument with an electrospray source. Direct infusion of pigment extracts yielded noisy and irreproducible spectra; therefore liquid chromatography mass spectrometry (LCMS) was used, employing a Dionex UltiMate 3000 high-performance liquid chromatography (HPLC) apparatus flowing at 100 µL/min plumbed to the electrospray source. Reversed-phase HPLC using a 2 mm × 10 cm C18 microbore column (5 micron particle size, 300A pore size, Upchurch) was developed over a 26 min gradient from 0% to 80% acetonitrile in 0.1% formic acid. Parent ions for fragmentation were chosen from the LCMS data, and collision-induced fragmentation spectra (including MSMS in which the parent ion is fragmented and MS^n^ in which the fragments are subsequently fragmented further, using normalized collision energies between 30% and 50%) were collected via direct infusion of the extracts in 50% methanol 1% formic acid.

### Chromatographic Separation of Pigments

Liquid chromatography was conducted as in the LCMS experiments but with the eluant collected in tubes (instead of flowing into the mass spectrometer) to allow visual and thin layer chromatography (TLC) inspection of pigments within fractions containing 50–100 µL of eluant. For TLC, collected fractions were concentrated to ~10 µL that was then applied in a line to a silica gel plate. Chromatography was then developed using 3:1 phenol:water, as done by Nijhout (Nijhout 1997).

### Redox Color Dependence

Extracted pigments were chemically reduced by adding a stock solution of ascorbic acid in water to achieve a final concentration of 1% w/v. The color change was quantified by scanning the absorbance of each sample from 400 to 700 nm using a Biotec Hybrid plate reader.

## Results

### Identification of Seven Pigments, Including Three Novel Pigments

**Fig. 1.**
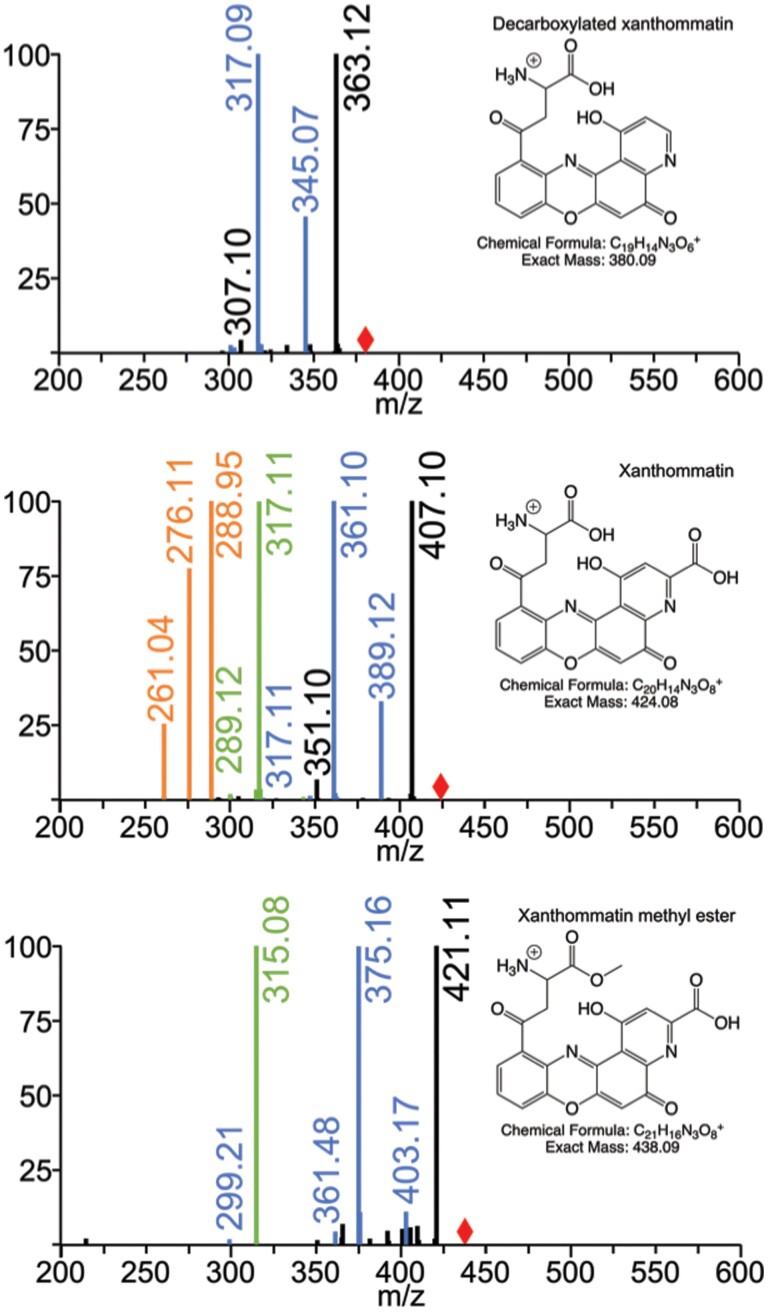
Fragmentation MS identifies decarboxylated xanthommatin, xanthommatin, and xanthommatin methyl ester. The positions of unfragmented parent ions are indicated with the red diamonds. Tandem mass spectrometry (black signal peaks) and MS^3^ (blue signal) of the parent [M + H] ion at 380 m/z (top panel) show fragments of 363, 345, 317, and 307 m/z, identifying this compound as decarboxylated xanthommatin. Tandem mass spectrometry (black), MS^3^ (blue), MS^4^ (green), and MS^5^ (orange) of the parent [M + H] ion at 424 m/z (middle panel) show fragments of 407, 389, 361, 351, 317, 289, 276, and 261 m/z, identifying this compound as xanthommatin. Tandem mass spectrometry (black) and MS^3^ (blue) of the parent [M + H] ion at 438 m/z (bottom panel) show fragments of 421, 403, 375, 361, 315, and 299 m/z, identifying this compound as xanthommatin methyl ester. (Fragmentations commonly seen in electrospray ionization mass spectrometry that informed these identifications are summarized by Ma et al. 2014).

**Fig. 2.**
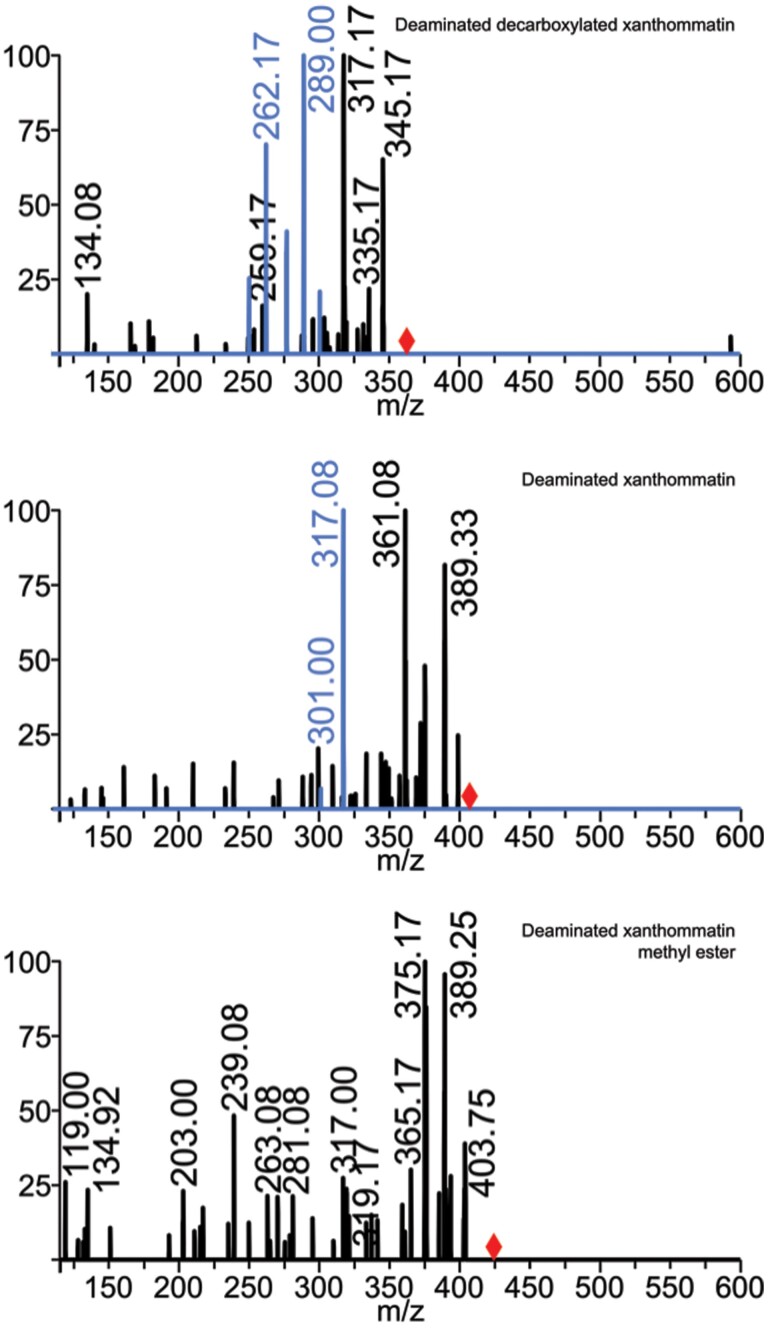
Fragmentation MS suggests the novel compounds deaminated decarboxylated xanthommatin, deaminated xanthommatin, and deaminated xanthommatin methyl ester. The positions of unfragmented parent ions are indicated with the red diamonds. Tandem mass spectrometry (black signal) and MS^3^ (blue) of the parent [M + H] ion at 363 m/z (top panel) show fragments of 345 and 317 m/z, suggesting this compound is deaminated decarboxylated xanthommatin. Tandem mass spectrometry (black) and MS^3^ (blue) of the parent [M + H] ion at 407 m/z (middle panel) show fragments of 389, 375, 361, and 317 m/z, suggesting this compound is deaminated xanthommatin. Tandem mass spectrometry of the parent [M + H] ion at 421 m/z (bottom panel) shows fragments of 403, 389, 375, 365, and 317 m/z, suggesting this compound is deaminated xanthommatin methyl ester.

High-performance liquid chromatography enabled mass spectrometric observation of seven candidate pigment compounds extracted from the orange sections of *D. plexippus* wings, with correlation to pigments visualized in TLC. The initial manifestations of different pigments in the liquid chromatography as seen by TLC of eluting fractions are referred to in Table 1. As shown in Fig. 1, using the mass-to-charge-ratio (m/z) values observed via LCMS for three of these candidates as parent ions in fragmentation experiments employing MSMS and MS^n^, we were able to identify these three compounds as decarboxylated xanthommatin, xanthommatin, and xanthommatin methyl ester. Three additional pigment compounds were consistent with deaminated forms of these three amine-containing pigments (Fig. 2). The exact chemical structure of these deaminated pigments has not been unambiguously established. These putative deaminated forms were not created by in-source decay, since they had different elution times from the corresponding aminated compounds. Furthermore, none of these putative deaminated compounds were seen in monarch eye extracts despite seeing a strong signal for xanthommatin (Supplementary Material Section 1). The deaminated pigments that we are proposing have not been previously described in any organism.

**Table 1.** Initial LC appearances of different pigments as seen by TLC of eluting fractions

Pigment color	R*f* upon TLC	Collected LC fraction	Corresponding LCMS peak	Observed mass	Compound(s)
Yellow	0.599 ± 0.060	13–13.5 min	13.47 min	380 m/z	decarboxylated xanthommatin
Orange	0.376 ± 0.025	13.5–14 min	13.99 min	424 m/z	xanthommatin
*	*	14–14.5 min	14.24 min	438 m/z	xanthommatin methyl ester
Yellow	0.426 ± 0.015	15–16 min	15.41 min 15.54 min	363 m/z 439 m/z	deam-dc-xanth α-hyd-xanth Me
Orange	0.450 ± 0.022	16–17 min	16.65 min 16.67 min	421 m/z 407 m/z	deam-xanth Me deam-xanth

Pigments are referred to in the order of their elution (initial appearance) from the HPLC. Due to tailing of the HPLC peaks, the first pigment has not disappeared before the second pigment appears, and so on. ‘Pigment color’ refers to a by-eye estimation of the hue, and real differentiation of the pigments is shown in their different migration upon TLC. TLC migration of xanthommatin agrees well with that observed by Nijhout et al (1997). The asterisk (*) denotes that no new TLC band appears in the 14–14.5 min fraction, and the orange band at R*f* = 0.376 persists in this fraction (perhaps obscuring the less abundant xanthommatin methyl ester). Corresponding LCMS peaks from extract ion chromatograms for each of the observed m/z molecular ions are indicated by the elution times at their maximum abundance. (Such peaks rise sharply—in a matter of seconds—and then tail off over a period of about a minute.) ‘Deam-’ indicates deaminated compounds, with ‘dc-xanth’ referring to decarboxylated xanthommatin, ‘xanth Me’ referring to xanthommatin methyl ester, ‘xanth’ referring to xanthommatin, and ‘α-hyd-xanth Me’ referring to α-hydroxy-xanthommatin methyl ester. Identification of compounds was accomplished by fragmentation MS as shown in Figs. 1 and 2.

An additional candidate pigment compound was observed at a peak elution time of 15.54 min having a mass of 439 m/z (as referred to in Table 1). This mass corresponds to one observed by Panettieri et al. (2018), which they assigned to the compound α-hydroxy-xanthommatin methyl ester. We were unable to fragment this molecular ion due to it having the same parent mass as the xanthommatin methyl ester isotope bearing a single atom of ^13^C; but we note that it does elute separately from the xanthommatin methyl ester and may indeed be the compound suggested by Panettieri et al.

### Relative Abundances of the Pigments

**Fig. 3.**
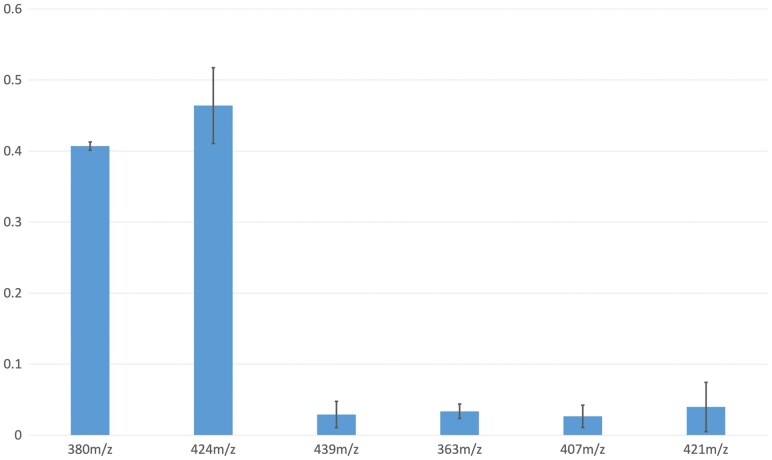
Relative amount of pigments. The areas underneath peaks identified in extract ion chromatograms of LCMS runs of extracts were determined and then normalized to the total signal of the six pigments shown, drawing data from duplicate experiments. The y-axis is the fraction of this total signal. The compound at 380 m/z is decarboxylated xanthommatin; that at 424 m/z is xanthommatin; signal at 439 m/z (elution peak at 15.5 min) is believed to be from α-hydroxy-xanthommatin methyl ester; and the compounds at 363, 407, and 421 m/z are putative deaminated forms of decarboxylated xanthommatin, xanthommatin, and xanthommatin methyl ester respectively. Signal at 438 m/z (elution peak at 14.2 min) is xanthommatin methyl ester and varied between 0.08 and 0.95 abundance as a fraction of the total signal from the other six pigment compounds.

LCMS allowed relative quantitation of compounds, which LCMSMS (online automated fragmentation during chromatography) would not have afforded. Comparisons of peak areas from extract ion chromatograms enabled the relative quantitation of each of the seven pigment compounds (Fig. 3). The most abundant pigment compound is xanthommatin, with decarboxylated xanthommatin only ~12% less abundant than the carboxylated form. Xanthommatin methyl ester abundance varied from run to run, depending on the time since the sample had been extracted. As discussed below, this may indicate methylation post extraction. The α-hydroxy-xanthommatin methyl ester is significantly less abundant than either the xanthommatin or decarboxylated xanthommatin, at less than 7% of the abundance of the unmodified ommatin pigment. Though quadrupole ion trap mass spectrometry has a somewhat limited linear range of detection and quantitative comparisons can be additionally complicated by the differences in ionization efficiencies between various compounds, these compounds are largely similar in structure and chemistry, so their relative abundance comparisons are meaningful.

The putative deaminated compounds might be assumed to be somewhat less similar in ionization efficiency when compared to the aminated forms (since the amine is the most likely moiety in these compounds to bear the adducting proton that gives charge to the molecular ions); therefore caution should be exercised in evaluating the comparative abundances between the aminated and putative deaminated forms in quantitative terms. Nonetheless, the putative deaminated compounds are likely much less abundant than their aminated correlates (as these data suggest); and confidence can be had in the comparisons between abundances of the three putative deaminated forms. Though the most abundant of these compounds gives less than 4% of the signal of unmodified xanthommatin, the three putative deaminated forms are all within one standard deviation of each other in abundance.

### Redox Color Variation

**Fig. 4.**
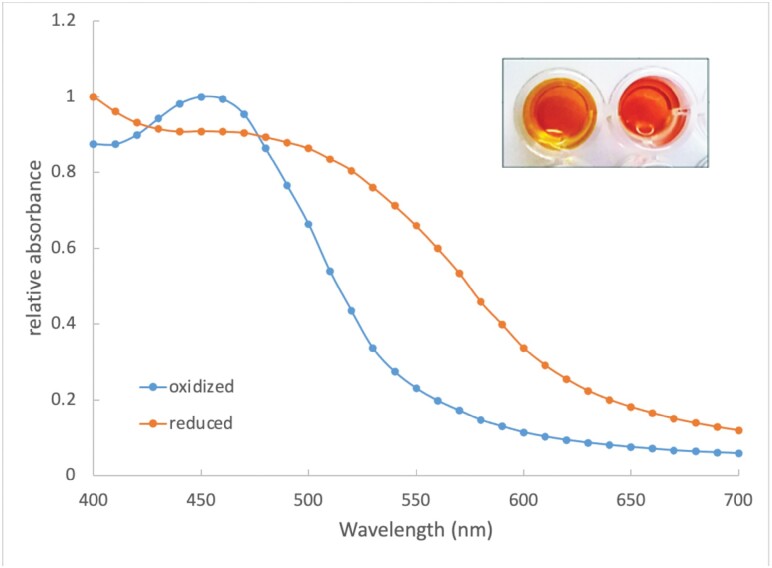
Redox states and colors. Extract was reduced using 1% ascorbic acid, which changed the color of the extract from yellow to red (inset photo; oxidized on left, reduced on right). Spectrophotometric scans of the visible-light-wavelength region show a prominent absorption peak of the oxidized extract at 450 nm which is greatly shifted to higher wavelengths in the chemically reduced extract.

Color of the pigment extracts differs depending on the redox state, with the oxidized pigments being more yellow and the reduced pigments more red (Fig. 4). Liquid chromatography mass spectrometry of the reduced pigment extract showed a substantial diminution of signal for the xanthommatin, decarboxylated xanthommatin, and xanthommatin methyl ester compounds; with a concomitant appearance of signal for the dihydroxanthommatin versions of these molecules, differing by the addition of two atoms of hydrogen. Supplementary Material Section 2 shows the fragmentation data confirming the identification of these reduced molecules.

## Discussion

In view of the importance of *D. plexippus* as a species, discovering the wing pigments that give this organism its familiar orange hue seems meaningful. We are unaware of previous determinations of the biochemical basis of monarch wing color.

**Fig. 5.**
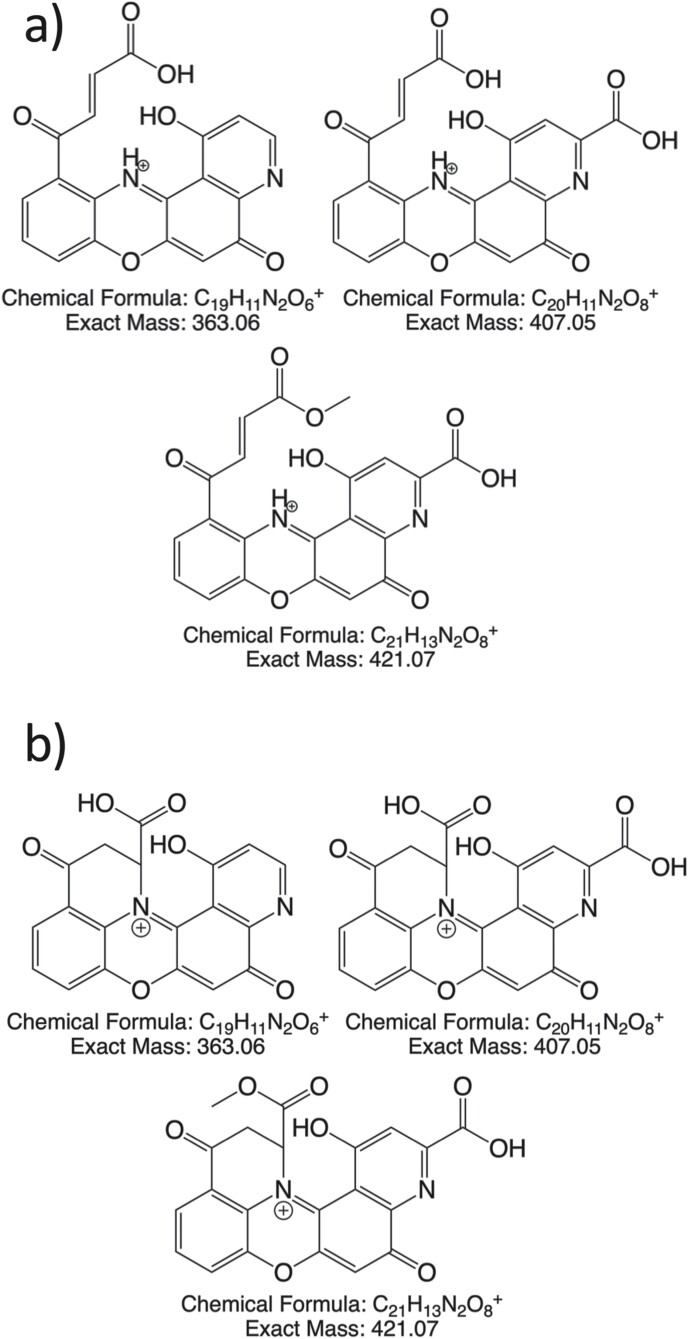
Proposed deaminated ommatin structures. Possible structures for the compounds deaminated decarboxylated xanthommatin (363 m/z), deaminated xanthommatin (407 m/z), and deaminated xanthommatin methyl ester (421 m/z) might result from a Hoffman-type elimination leaving a carbon-carbon double bond (panel a) or from a cyclization (panel b) analogous to the formation of pyroglutamic acid from glutamine.

Ommochromes have been previously reported in a variety of arthropods (Wiley and Forrest 1981, Riou and Christidès 2010, Shamim et al. 2014, Futahashi and Osanai-Futahashi 2021). Alongside the well-known and well-characterized xanthommatin (see MassBank of North America 2022; PubChem 2022) which is the most abundant pigment we observe, we find several modified ommatins including decarboxylated xanthommatin and xanthommatin methyl ester, both previously observed by Panettieri et al. (2018) in other nymphalid butterflies that mimic *Danaus* species other than *plexippus*. (A full discussion of the comparison between compounds identified in the present work with compounds identified by Panettieri et al is given in Supplementary Material Section 3). Additionally, we observe three molecular masses that we attribute to novel deaminated xanthommatin derivatives, the structures of which have not yet been determined in detail. These deaminated compounds could result from either of two types of modification of the known ommatins. They may result from elimination reactions from these previously observed compounds, yielding alkene products as seen in a Hofmann elimination reaction (Hofmann 1851) as shown in Fig. 5a. Alternatively, deamination may take place via a cyclization as is seen in the formation of pyroglutamic acid from amino terminal glutamine (Kumar and Bachhawat 2012), which would create structures as shown in Fig. 5b. We are unaware of previously discovered biological Hoffman-type deamination reactions and would therefore propose the cyclic structures to be more likely. Further work will be necessary to confirm which of the proposed structures are correct and to identify the enzymes that catalyze the deaminations. We are confident that these deamination modifications were not caused during extraction or via in-source fragmentation during LCMS as evidenced by their absence in extracts of butterfly eyes that contain abundant xanthommatin (Supplementary Material Section 1). Methylation, on the other hand, does appear to happen within the extracts, as we see higher amounts of the xanthommatin methyl ester the longer a sample sits in the acidic methanol used to extract the pigments. We note that we do not see methylation of the other observed ommatins besides xanthommatin, nor do we find any unmethylated α-hydroxy-xanthommatin methyl ester. Our presumption is that there is some xanthommatin methyl ester to be found in vivo, but that more is produced by ongoing adventitious methylation post extraction.

Our finding that the pigments that we observe in *D. plexippus* wings change in color upon being chemically reduced implies that these butterflies may control redox state in order to vary coloration. Indeed, we note that different scales within the same wing section differ in color hue, which may reflect differences in redox state from one scale to another. An alternate possibility is that different wing scales contain different mixtures of the pigments observed, thus varying scale coloration. Conceivably future work could involve investigations of individual wing scales, perhaps using Matrix-Assisted Laser Desorption Ionization mass spectrometry, in order to look at the relative abundances of the different pigments within scales themselves.

## Supplementary Material

ieac076_suppl_Supplementary_Material_Section_1

ieac076_suppl_Supplementary_Material_Section_2

ieac076_suppl_Supplementary_Material_Section_3
