# Supplementary material for: Ommochrome Wing Pigments in the Monarch Butterfly Danaus plexippus (Lepidoptera: Nymphalidae)
Source: J Insect Sci. 2022 Dec 23;22(6):12. doi: 10.1093/jisesa/ieac076 (PMC9780745; doi:10.1093/jisesa/ieac076)
Supplement: ieac076_suppl_Supplementary_Material_Section_1 [file ieac076_suppl_supplementary_material_section_1.pdf]

Supplementary Material Section 1: Comparison of pigment abundances from wings and eyes.

Compound mass	Pigment compound	Abundance in wing	Abundance in eye
380 m/z	dc-xanth	43.8	10.2
424 m/z	xanthommatin	47.1	89.7
439 m/z	α-hyd-xanth Me	1.8	0.0
363 m/z	deam-dc-xanth	2.4	0.0
407 m/z	deam-xanth	3.6	0.0
421 m/z	deam-xanth Me	1.3	0.0

Side-by-side LCMS analyses were run for extracts of orange wing sections and dissected whole eyes, with peak abundance given as percent of total signal for these six peaks. Compounds were identified as described in main text. “Deam-“ indicates deaminated compounds, with “dc-xanth” referring to decarboxylated xanthommatin, “xanth Me” referring to xanthommatin methyl ester, “xanth” referring to xanthommatin, and “α-hyd-xanth Me” referring to α-hydroxy-xanthommatin methyl ester.
