# Supplementary material for: Ommochrome Wing Pigments in the Monarch Butterfly Danaus plexippus (Lepidoptera: Nymphalidae)
Source: J Insect Sci. 2022 Dec 23;22(6):12. doi: 10.1093/jisesa/ieac076 (PMC9780745; doi:10.1093/jisesa/ieac076)
Supplement: ieac076_suppl_Supplementary_Material_Section_2 [file ieac076_suppl_supplementary_material_section_2.pdf]

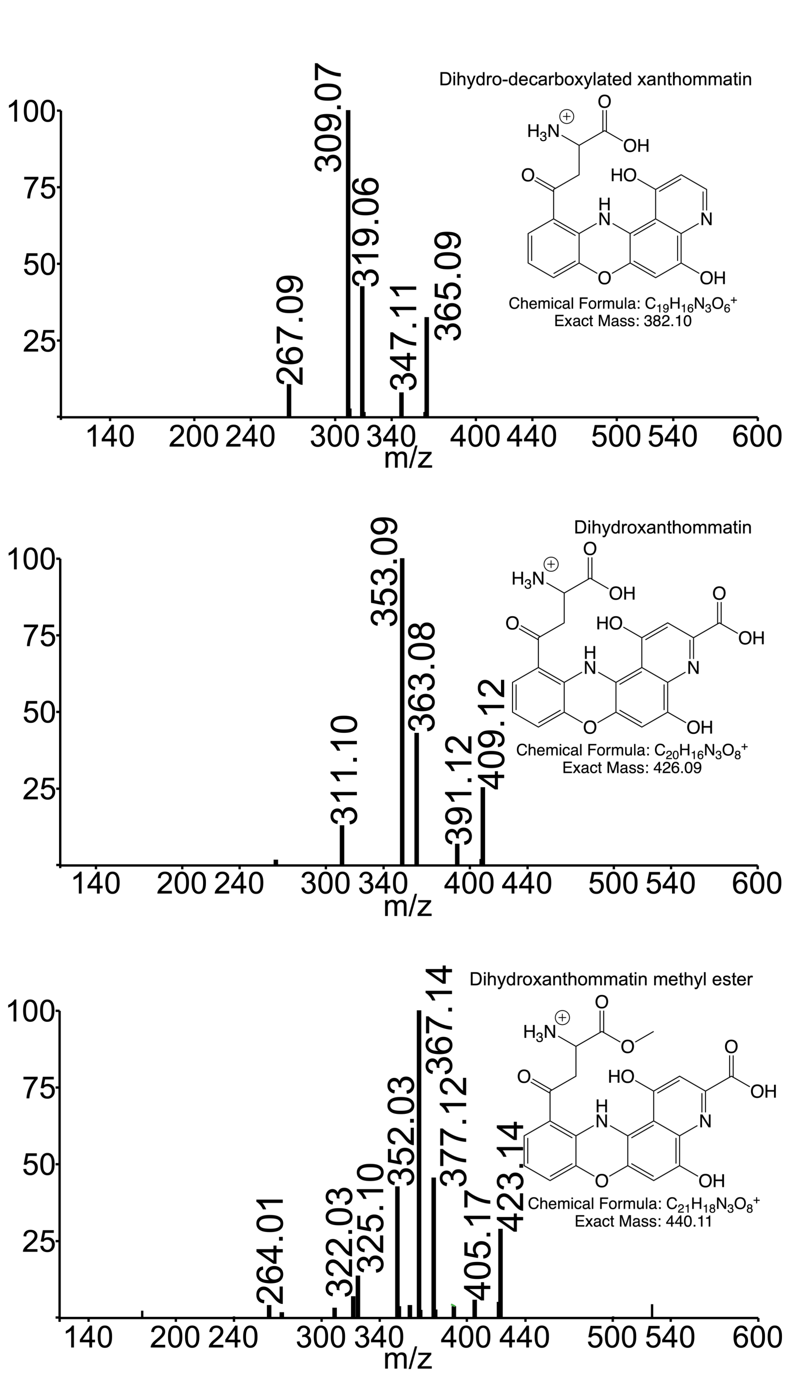

Supplementary Material Section 2: Fragmentation MS identifies reduced dihydro- versions of decarboxylated xanthommatin, xanthommatin, and xanthommatin methyl ester. Monarch pigments from orange wing sections were extracted and analyzed as detailed in Materials and Methods. Tandem mass spectrometry of the parent [M+H] ion at 382 m/z (top panel) show fragments of 365, 347, 319, and 309 m/z, identifying this compound as dihydro-decarboxylated xanthommatin. Tandem mass spectrometry of the parent [M+H] ion at 426 m/z (middle panel) show fragments of 409, 391, 363 and 353 m/z, identifying this compound as dihydroxanthommatin. Tandem mass spectrometry of the parent [M+H] ion at 440 m/z (bottom panel) show fragments of 423, 405 and 377 m/z, identifying this compound as dihydroxanthommatin methyl ester.
