# Supplementary material for: Ommochrome Wing Pigments in the Monarch Butterfly Danaus plexippus (Lepidoptera: Nymphalidae)
Source: J Insect Sci. 2022 Dec 23;22(6):12. doi: 10.1093/jisesa/ieac076 (PMC9780745; doi:10.1093/jisesa/ieac076)
Supplement: ieac076_suppl_Supplementary_Material_Section_3 [file ieac076_suppl_supplementary_material_section_3.pdf]

Section 3: Detailed comparison between pigments identified in this study and those previously identified in the study of *Elymnias hypermnestra* (Lepidoptera: Nymphalidae)

Panettieri et al. (2018) reported nine separate pigments – all ommatins – in their investigations of other nymphalid butterflies, only two of which (xanthommatin and decarboxylated xanthommatin) had been previously reported in the literature. We see evidence for four of the nine reported compounds (including the two identified prior to Panettieri's work) in our studies of the pigments from *D. plexippus* wings.

The Panettieri group observed two isomers of m/z 424 that eluted separately upon HPLC. (All masses in this discussion are the m/z ratios for the singly charged [M+H] molecular ions.) One of these isomers was found only in *Elymnias hypermenestra tinctoria* while the other was common to both *E. h. tinctoria* and *E. h. baliensis*. The compound attributed uniquely to *E. h. tinctoria* was purported to be xanthommatin, and that common to both subspecies was named elymniommatin (which the authors proposed as a pair of isomeric structures because of not being able to distinguish which of the rings in the phenoxazine system the amine-containing side-chain was attached to). We believe that it is more likely that the compound unique to *E. h. tinctoria* has the structure referred to as elymniommatin (or iso-elymniommatin), and that the structure common to both subspecies of *E. hypermenestra* – as well as seen by us in *D. plexippus* – is xanthommatin. We base this conjecture on the similarities of the tandem MS fragmentation pattern given by Panettieri et al (said to be elymniommatin) not only with our own data (shown in Fig. 1 of our paper), but also with the fragmentation data given in both PubChem (PubChem 2022) and MoNA ("MassBank of North America" 2022) for

xanthommatin. Briefly, in all of these fragmentation MS data, xanthommatin is shown to give a predominant fragment peak at 407 with additional salient fragments at 361, 351, and 317 (with all but the PubChem spectrum also showing a fragment at 389). The predominant fragment at 407 results from the loss of the amine, which amine is not present in the proposed structure of elymniommatin. In contrast, the MSMS spectrum presumed by Panettieri et al to be that of xanthommatin shows its most prominent peak at 361 with other conspicuous peaks at 317 and 307, and a small unlabeled peak that might be at 407. The fragment at 307 is the only peak of any size that is unique among these various data, and is found only in the Panettieri spectrum proposed to be that of xanthommatin. In view of all of these data, we conclude that the fragmentation pattern attributed to xanthommatin by Panettieri et al is likely that of another related compound – probably having the structure proposed for elymniommatin – while the isomer that they have presumed to be elymniommatin is likely actually xanthommatin.

It should be noted that the structure of xanthommatin was discovered many decades ago by synthesizing the molecule (Butenandt et al. 1954), and has been extensively studied including via mass spectrometry (PubChem 2022, "MassBank of North America" 2022).

Panettieri et al reported isomers at m/z 380, including decarboxylated xanthommatin (which they found to be unique to *E. h. baliensis*) and a compound they named tinctoriommatin (unique to *E. h. tinctoria*, and also proposed as a pair of isomeric structures because of not being able to distinguish which of the rings in the phenoxazine system the amide-containing side-chain was attached to). Our fragmentation MS of decarboxylated xanthommatin (see Fig. 1) shows a prominent peak at 363, with strong

peaks at 345 and 317, and minor peaks at 334 and 307. The Panettieri fragmentation data for decarboxylated xanthommatin also shows a prominent peak at 363 with peaks at 345, 334, 317, and 307. Their fragmentation data for trinctoriommatin/iso-tinctoriommatin show the prominent peak to be at 317 with an additional peak at 307 and smaller peaks at 291 and 261. Our data therefore are in good agreement with the identification of decarboxylated xanthommatin as reported by Panettieri et al.

The compound having a mass of 438 was first observed by Panettieri et al, which compound they named xanthommatin methyl ester. We also observe this compound in our extracts from *D. plexippus* wings. Panettieri et al spell out the great care that they took in trying to avoid modification of pigments after extraction, but they (like we) did employ acidic methanol as the extraction solvent. Our discussion of the possible methylation of xanthommatin post-extraction is given in the body of our paper.

Panettieri et al did not report any of the deaminated ommatins that we postulate, with the single exception that they did assume deamination – with concomitant introduction of a hydroxyl – in the α-hydroxy-xanthommatin methyl ester (at mz 439). Though we also observed a compound at this molecular weight, we were unable to accomplish fragmentation MS of it in order to confirm its structure, and we can only assume that it might be the compound reported by Panettieri et al.
